# The effect of modified locking methods and suture materials on Zone II flexor tendon repair—An *ex vivo* study

**DOI:** 10.1371/journal.pone.0205121

**Published:** 2018-10-05

**Authors:** Susumu Yoneda, Hirotaka Okubo, Stephen W. Linderman, Nozomu Kusano, Matthew J. Silva, Stavros Thomopoulos, Fuminori Kanaya, Richard H. Gelberman

**Affiliations:** 1 Department of Orthopaedic Surgery, Washington University, St. Louis, Missouri, United States of America; 2 Department of Orthopedic Surgery, Graduate School of Medicine, University of the Ryukyus, Okinawa, Japan; 3 Department of Orthopedic Surgery, Tominaga-Kusano Hospital, Niigata, Japan; 4 Department of Orthopedic Surgery, Columbia University, New York, New York, United States of America; 5 Department of Biomedical Engineering, Columbia University, New York, New York, United States of America; Mayo Clinic Minnesota, UNITED STATES

## Abstract

The failure rate of intrasynovial tendon repair is high due to substantial elongation at the repair site and to the development of adhesions between the tendon’s surface and the surrounding digital sheath. To minimize these complications, we sought to reduce the incidence of gapping and to facilitate the initiation of early motion by improving the time zero structural properties of repair. The Winters-Gelberman 8-strand repair technique was modified by adding surface lock loops and by using Fiberwire suture material. Forty-eight canine flexor digitorum profundus tendons were transected and repaired with one of three 8-strand techniques (Pennington modified Kessler, half hitch loops, or surface locking Kessler) using either 3–0 Supramid or 4–0 Fiberwire suture. Biomechanical testing was performed to determine the physiologic and failure mode properties of the repairs. The surface locking Kessler technique improved repair maximum load, load necessary to create a 2 mm repair site gap, and yield force compared to the modified Kessler and half hitch loop techniques. Fiberwire suture improved maximum load, the load necessary to create a 2 mm repair site gap, stiffness, and yield force compared to Supramid suture. Failure occurred by both suture pull out and by suture breakage in the modified Kessler, Supramid suture repair group. Failure occurred consistently by suture breakage in the surface locking Kessler, Supramid suture repair group. These results reveal that a novel locking Kessler repair is significantly stronger than the current state-of-the art flexor tendon suture repair technique. The use of a surface locking Kessler technique with Fiberwire suture markedly improves the mechanical properties of intrasynovial tendon repair by reducing the risk of post-operative gapping and rupture.

## Introduction

The outcomes of intrasynovial flexor tendon repair are highly variable due to the frequent occurrence of repair site elongation and catastrophic rupture and due to adhesion formation between the tendon and its surrounding digital sheath [1,2]. Two approaches have been employed to reduce the incidence of these complications: improved techniques for repairing tendon to reduce gap formation and rupture, and early controlled motion rehabilitation to prevent adhesion formation. The objective of the current *ex vivo* study was to explore a method for improving the initial structural properties of intrasynovial tendon repair, thereby reducing the incidence of gap formation in the early stages following tendon suture.

*In vivo* animal studies indicate that, under conditions of early controlled mobilization, the strength values achieved at time zero persist throughout the initial four weeks post repair [3]. The repair site does not accrue additional strength beyond that of the time zero repair until four to six weeks post repair. Thus, the achievement of a stiff and strong initial repair is of critical importance to the initiation of early controlled mobilization and to achieving longer term positive clinical outcomes.

The initial mechanical strength of a repaired tendon depends on suture strand number, core suture purchase length, anchoring technique, lock diameter, and core suture material properties [4,5]. Recently, we reported that the failure mode of the 8-strand half hitch loop technique (HHL) with 3–0 Supramid suture (Supramid Extra II; S. Jackson Inc., Alexandria, VA) was primarily suture breakage. In contrast, the 8-strand Winters-Gelberman grasping technique with 3–0 Supramid failed primarily by suture pullout [6]. Under conditions where suture pullout is the primary mode of failure, the provision of stronger suture material has little effect on the mechanical properties of repair [7]. Thus, we were motivated to explore a modified locking technique with a stronger suture material in order to increase pull out force, improving repair site strength and stiffness. As 4–0 Fiberwire (Arthrex, Naples, FL) is reportedly stronger than 3–0 Supramid [8, 9], we hypothesized that a secure locking technique with 4–0 Fiberwire suture would improve the initial mechanical properties of flexor tendon repair compared to current techniques.

The aim of this *ex vivo* biomechanical study was to improve the time zero mechanical properties of flexor tendon repair by employing a new locking technique and improved suture properties in order to reduce the incidence of gap formation and the complications associated with suture failure.

## Materials and methods

### Study design

**Fig 1:**
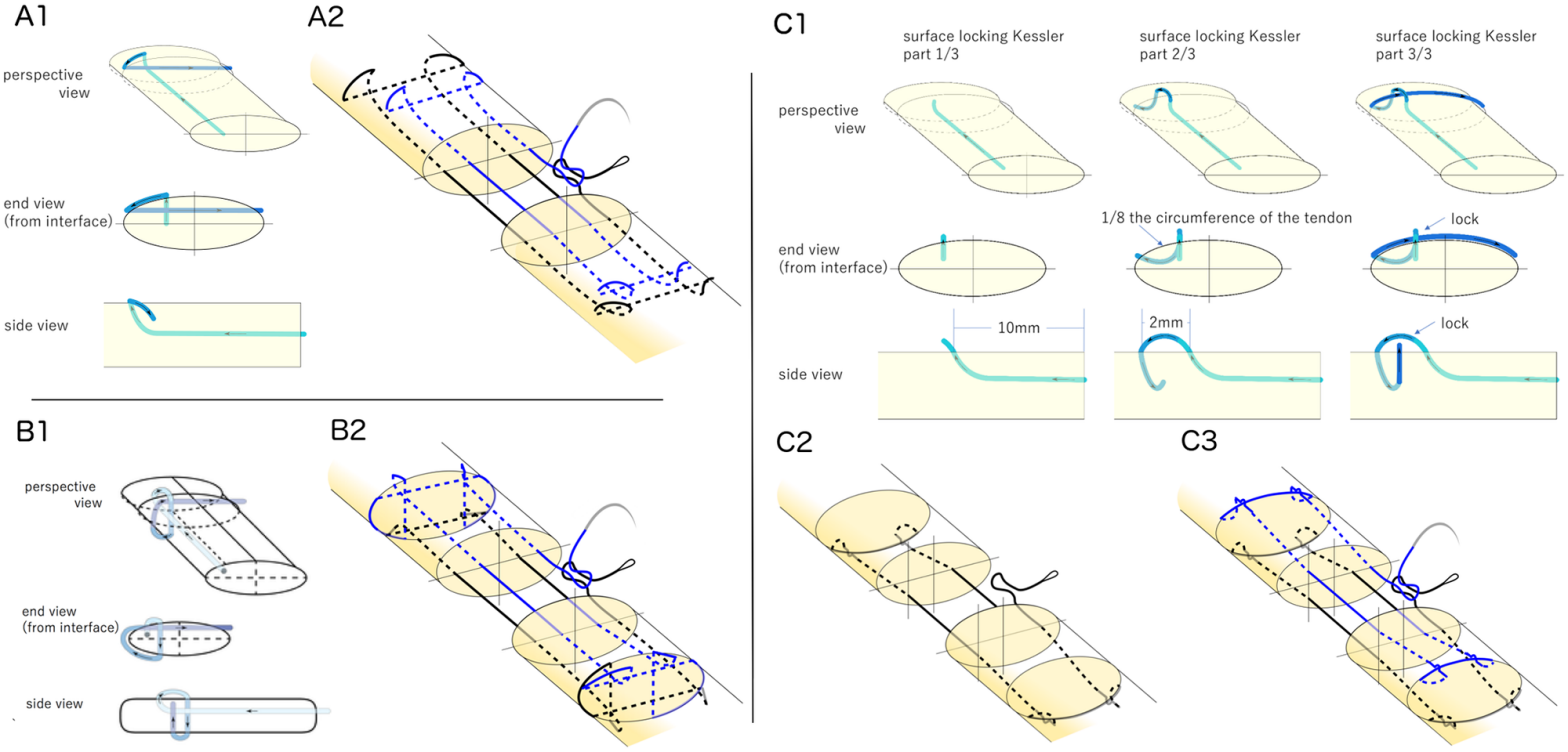
Locking and suture configurations are shown. (A1) Pennington modified Kessler (MK) locking loop. (A2) 8-strand with MK. Looped suture was used. This is a continuous piece of suture, where suture coloration is artificially added to aid in following the suture path. The first 4 strands are drawn in black and the others are drawn in blue. (B1) Single hitch loop (adapted from [6]). (B2) 8-strand using half hitch loop. See detail in [6]. (C1) Surface locking Kessler (SLK) loop. The two suture passes required to make a surface locking Kessler loop are shown sequentially as an end view from the transection interface, a side view, and a perspective view. The locking diameter is one eighth of tendon. Transverse suture is securely locked by the longitudinal suture. (C2) The first four passes were performed on the dorsal side. (C3) 8-strand using SLK.

In this *ex vivo* study, 48 cadaveric canine flexor digitorum profundus tendons were used. All tendons tested in this study were harvested from the hind-paws of healthy female adult mongrel dogs 20–30 kg in weight (Covance Research, Princeton, NJ), taken postmortem from an unrelated project that was approved by the Animal Studies Committee, Office of the Vice Chancellor for Research, at Washington University in St. Louis for RHG’s Animal Approval Protocol #20140115. Regarding to method of sacrifice, the canines were sedated with Propofol (6−7 mg/kg) and then given an overdose of Pentobarbital (greater than 150 mg/kg IV). The hind-paws were stored at -20 °C and thawed at 4 °C overnight before testing. The tendons were divided into 6 groups of 8 tendons each (Table 1). Prior to tendon transection, 5 lines were created around the repair (at the transection site, grasping sites on the either side of the transection level [12 mm], and 2 mm on either side of the repair site) to facilitate consistent suture purchase. Tendons were transected in Zone II, midway between the A2 and A4 pulleys. Sharp transverse lacerations were created and repaired under loupe magnification by one hand surgeon (SY). The suture material consisted of 3–0 Supramid or 4–0 Fiberwire (FiberLoop; Arthrex, Naples, FL). All sutures were used in looped configuration so that each pass resulted in two strands. The following repairs were performed using both Supramid and Fiberwire suture: (1) 8-strand modified Kessler (MK), (2) 8-strand HHL, (3) 8-strand surface locking Kessler (SLK) (Fig 1). The 8-strand MK was modified from the Winters-Gelberman 8-strand repair by using the Pennington locking loop to replace the original grasping loop. The 8-strand HHL was described in a previous study by Kormpakis et al [6]. The SLK repair was modified from the Winters Gelberman 8-strand repair by using SLK locking loops [10]. The initial 4 passes were performed on the dorsal side of the tendon and the subsequent four passes on the volar side, similar to the HHL repair. The purchase of the SLK suture was 10 mm and the longitudinal locking length was 2 mm. For all repairs, a running peripheral suture (5–0 Prolene; Ethicon, Somerville, NJ) with 2 mm purchase was used. As mild tension across the repair site improves gap force, we added and equalized tension across the repair site by measuring shortening [11]. Two transverse lines were drawn 12 mm proximal and distal to the transection sight prior to cutting the tendon. After repair, the distance between the lines was measured.

**Table 1:** Flexor tendon suture configurations.

Technique	Suture	Sample size
**modified Kessler**	3-0 Supramid	8
**half hitch loop**	3-0 Supramid	8
**surface locking Kessler**	3-0 Supramid	8
**modified Kessler**	4-0 Fiberwire	8
**half hitch loop**	4-0 Fiberwire	8
**surface locking Kessler**	4-0 Fiberwire	8

### Biomechanical testing

Repairs were tested biomechanically using methods described previously [4, 6, 12, 13]. The proximal tendon segment was secured with a triangle-toothed grip and the distal phalanx was secured in a custom grip. After preconditioning, repaired tendons were pulled in uniaxial tension at 0.3 mm/s until failure on a material testing machine (5866; Instron Corp., Norwood, MA), and strain was tracked optically using video of the test. Failure mode (i.e., suture pullout, suture breakage, or knot failure) was recorded. The maximum load (N), load to create a 2 mm gap (N), stiffness (N/mm) (the slope of the linear portion of the load–deformation curve), strain at 20 N (%), yield force (N) and a modified version of resilience (N strain) (the area under the load–strain curve up to the yield point) were determined using a custom-written code in MATLAB (Natick, MA), as described previously [6]. Load to create a gap of 2 mm between tendon stump ends (a threshold level that leads to decreased repair strength and increased adhesions [14,15]) was calculated by tracking the displacement between points ± 2 mm from the repair site with code in MATLAB.

### Statistics

Groups were compared using two-way analysis of variance (ANOVA) and simple main effect with Bonferroni correction was examined as a priori comparison. An alpha level of p < 0.05 was set for statistical significance. Results were plotted as mean ± standard deviation (SD).

## Results

### Tendon segment shortening percentage

There were no statistically significant differences among the six repair groups (p > 0.05 for each comparison) in tendon segment shortening. Tendon segment shortening percentage was approximately 14.5% (S1 Dataset).

### Mechanical properties

#### Failure properties

**Fig 2:**
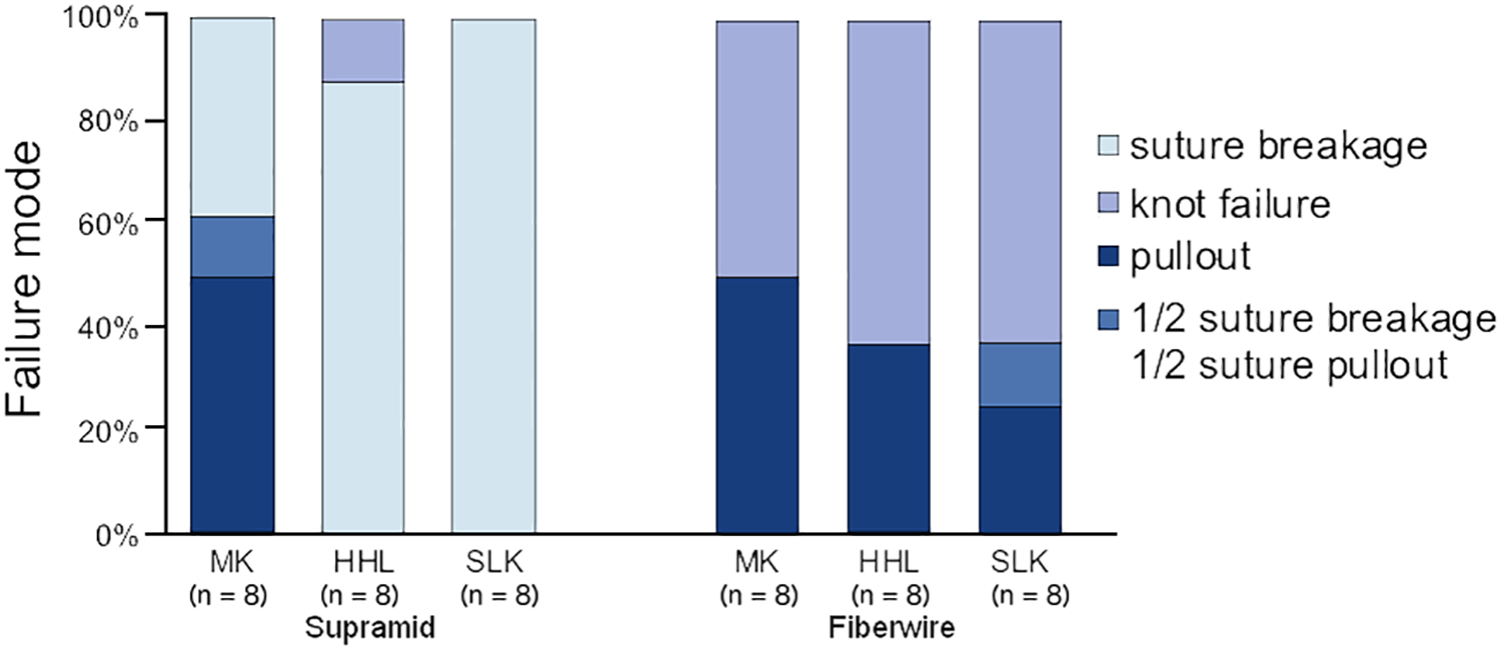
Failure mode. For Supramid suture, failure mode was by pullout/suture breakage in the MK repair group and by suture breakage in the HHL and SLK repair groups. For Fiberwire suture, there was an increase in knot failures and a decrease in pullout failures when comparing the MK to the HHL and SLK repairs. The HHL and SLK repairs with 3–0 Supramid failed by the limits of the suture strength. For the SLK repairs with 4–0 Fiberwire, the loops failed primarily by knot failure. (MK: modified Kessler, HHL: half hitch loop, SLK: surface locking Kessler).

**Fig 3:**
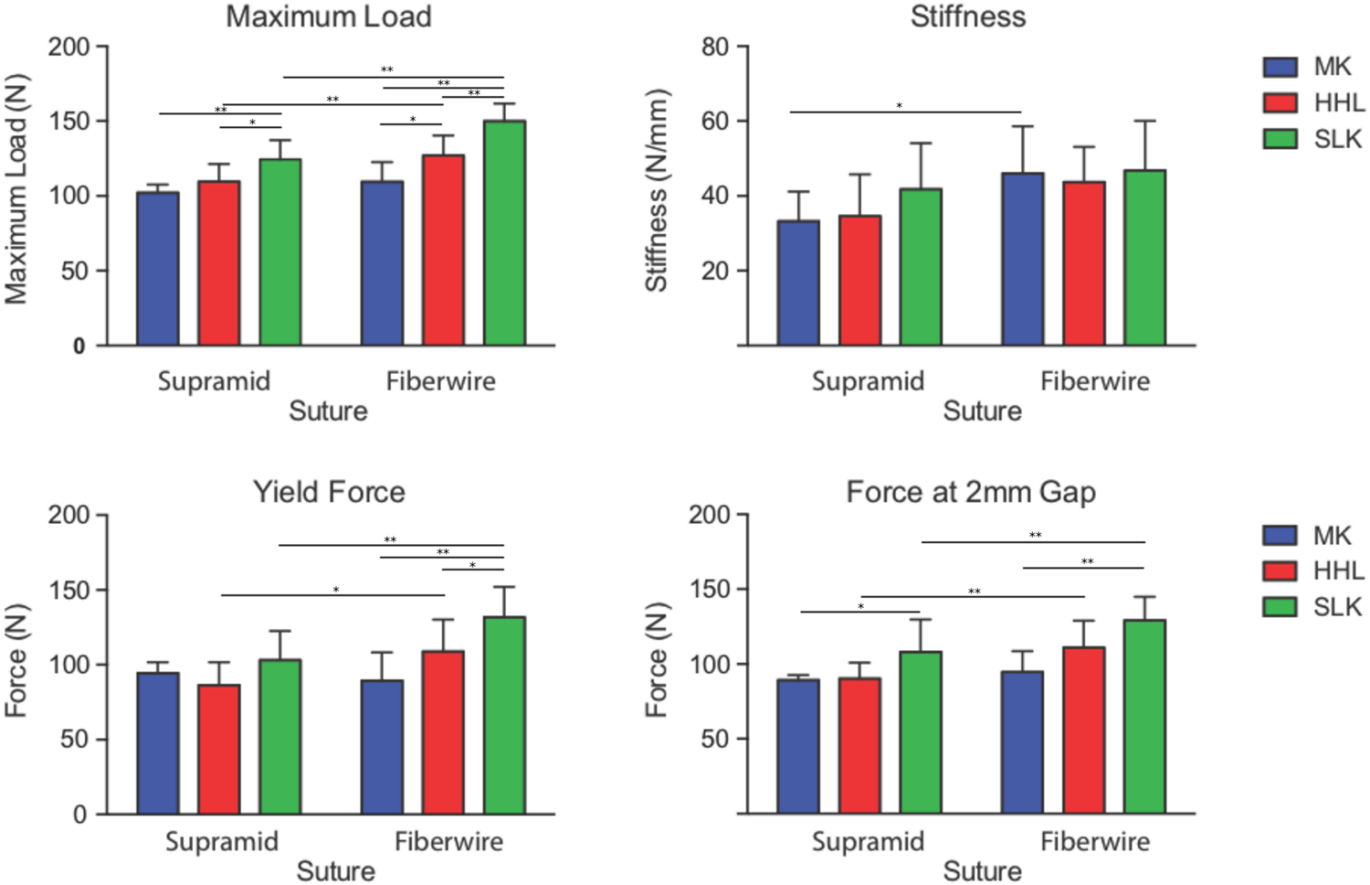
Mechanical properties. Maximum load, yield force, and 2 mm gap force were significantly increased for the SLK repairs compared to the MK repairs. All properties were higher for Fiberwire suture compared to Supramid suture. (MK: modified Kessler, HHL: half hitch loop, SLK: surface locking Kessler, * p < 0.05 **p<0.01 for priori comparison, mean ± standard deviations are shown).

Four of eight MK repairs with Supramid suture failed by suture pullout, one by a combination of pullout and suture breakage, and three by suture breakage. All of the HHL and SLK repairs with Supramid suture failed by suture breakage except one, which failed by suture pullout. When using Fiberwire suture, the failure modes of the repairs were both knot failure and suture pullout. (Fig 2). The SLK repairs with Fiberwire had the highest maximum load (with Fiberwire suture, MK: 109.4±13.1 N, HHL: 126.9±13.5 N, and SLK: 149.9±11.8 N) (Fig 3). When examining the effect of suture technique, the SLK repairs had significantly higher maximum load values than did the MK repairs (p<0.01) and HHL repairs (p<0.01). When examining the effect of suture material, Fiberwire had significantly higher maximum load than did Supramid (p<0.01). When examining the effect of suture material on each repair technique, Fiberwire had significantly higher maximum loads compared to Supramid in the HHL repairs (p<0.01) and SLK repairs (p<0.01), but did not differ in the MK repairs (p = 0.2).

#### Yield properties

The SLK repairs with Fiberwire had the highest levels of load to create a 2 mm gap (with Fiberwire suture, MK: 94.6±14.0 N, HHL: 110.9±18.1 N, and SLK: 129.1±15.7 N) and yield force (with Fiberwire suture, MK: 89.4±18.9 N, HHL: 108.9±21.4 N, and SLK: 131.8±20.3 N) (Fig 3). When examining the effect of suture technique, the SLK repairs had significantly higher loads to create a 2 mm gap and yield forces than did the other repairs (p<0.01 for each comparison). When examining the effect of suture material, Fiberwire had significantly higher loads to create a 2 mm gap and yield forces than did Supramid (p<0.01 for each comparison). When examining the effect of suture material on each repair technique, Fiberwire had significantly higher loads to create a 2 mm gap and yield forces compared to Supramid in the HHL repairs (p<0.01, p<0.05 respectively) and SLK repairs (p<0.01 for each comparison), but did not differ in the MK repairs (p = 0.49, p = 0.57 respectively). The results for strain at 20N and resilience can be found in the supplemental S1 Dataset.

#### Stiffness

While the SLK repairs with Fiberwire had the highest levels of stiffness overall (MK: 46.0±12.6 N/mm, HHL: 43.7±9.4 N, and SLK: 46.8±13.2 N), there was no significant effect of repair technique. When examining the effect of suture material, Fiberwire was significantly stiffer than Supramid (p<0.01).

## Discussion

The current experiment demonstrated that the surface locking Kessler modified tendon repair technique had significantly higher maximum loads, loads to create a 2 mm gap, and yield forces compared to the modified Kessler and the half hitch loop repairs. This study confirmed that, if the failure mode of a repair is suture pullout, further strengthening of the suture will not improve the mechanical properties of the repaired tendon [7]. Specifically, the MK repair with Supramid suture failed either by suture pullout and suture breakage. By increasing suture grasping, the HHL and SLK repairs with Supramid suture failed exclusively by suture breakage, demonstrating that the weak link of the repair was not the strength of the suture. Consistent with this premise, the use of the stronger Fiberwire suture in the HHL and SLK repairs improved the mechanical properties of the repair in a statistically and clinically significant manner.

Recent *in vivo* studies have demonstrated that the strength values achieved at time zero persist throughout the initial four weeks post tendon repair [3]. In an effort to improve the time zero structural properties of tendon repair, investigators have introduced a variety of technical modifications, the most promising of which have employed the addition of a tendon loop configuration to improve time zero strength [16]. Locking loops can fail, however, either by transecting tendon fibrils or by un-winding [17]. Un-winding can be initiated with less than 15 N of force for the most commonly employed technique, the modified Kessler method [18]. The technique’s transverse segment narrows as longitudinal forces are applied [19]. This phenomenon results in suture elongation and repair site gapping as the suture bends and un-winds [20]. As a result, the Kessler type configuration has been prone to failure. We modified this repair technique by introducing a surface lock [10] in order to increase pullout force by preventing suture un-winding, thereby allowing failure to be directly related to the strength of the suture. The SLK loop technique is similar in complexity to other multi-strand repair methods (e.g. MGH, Savage) [21, 22]. Passage of the dorsal suture is similar in configuration to the commonly performed modified Pennington suture and is similar in complexity to the dorsal portion of the SLK configuration. The volar suture is similar to the previously described Winters-Gelberman method [23].

A common reason for tendon rupture is the patient’s resumption of unrestricted grasping activities at an early stage following repair. The *in vivo* tendon forces generated during hand function are approximately 35 N and 60 N for normal [24] and strong [25] grasps, respectively. Based on a safety zone concept [26], the surgically repaired tendon should be capable of withstanding approximately 60 N for the initiation of controlled motion rehabilitation and 90 N for unrestricted grasp [27] at time zero. The modifications introduced here achieved an average of 149.9 N of *ex vivo* maximum load and 129.1 N in load to create a 2mm gap. These are the highest reported values for intrasynovial flexor tendon repair. Improved structural properties such as those seen here should reduce the incidence of rupture caused by unrestricted grasp activities and expand the safety zone required for the initiation of controlled motion rehabilitation.

There are several limitations to this study. First, as we utilized a noncyclic linear load-to-failure test model, the influences of cyclic or curvilinear loading conditions could not be addressed. Second, exposed suture and excessive suture crossing the repair site can increase friction and cause concerns regarding adhesion formation. However, prior *in vitro* studies demonstrate that the use of Fiberwire reduces resistance force [21, 28]. Under well controlled rehabilitation, the exposed suture for the performance of secure locking loops [24] and for the 8 strand configuration did not adversely affect the achievement tendon gliding and digital range of motion in prior *in vivo* studies [29]. Third, it is possible that 8-strand repairs with the SLK suture may cause injury to the tendon’s vascular supply due to the dorsal placement of some of the loops. However, dorsal placement has been shown to be well tolerated in a prior *in vivo* study [22]. Fourth, the use of Fiberwire may lead to higher rates of knot failure, as mentioned recently [27, 30]; improvements in knot technique may be required to address this [21].

In conclusion, the results of this study confirm our hypothesis that the SLK technique combined with the use of Fiberwire suture improves the initial mechanical properties of intrasynovial flexor tendon repair in a clinically relevant manner.

## Supporting information

S1 Dataset Data for each sample reported throughout this study. The first sheet provides the individual sample identifier, group type, shortening percentage, failure mode, maximum load, load to create a 2 mm gap, stiffness, modified resilience, strain at 20 N, yield force and displacement at yield force. The second sheet summarizes these data as mean, standard deviation, and sample size for each sample group and result.
(XLSX)
